# Histopathological characteristics are instrumental to distinguish monomorphic from polymorphic maculopapular cutaneous mastocytosis in children

**DOI:** 10.1111/ced.15262

**Published:** 2022-07-11

**Authors:** Maud A. W. Hermans, Suzanne G. M. A. Pasmans, Nicolette J. T. Arends, Thierry P. P. van den Bosch, Paul L. A. van Daele, Martijn B. A. van Doorn, Elise J. Huisman, Antien L. Mooyaart, Jeffrey Damman

**Affiliations:** ^1^ Section of Allergy and Clinical Immunology Department of Internal Medicine Erasmus University Medical Center Rotterdam The Netherlands; ^2^ Department of Dermatology Erasmus University MC Sophia Children's Hospital Rotterdam The Netherlands; ^3^ Section of Allergy Department of Paediatric Medicine Erasmus University MC Sophia Children's Hospital Rotterdam The Netherlands; ^4^ Department of Pathology Erasmus University MC Rotterdam The Netherlands; ^5^ Department of Paediatric Haematology Erasmus University MC Sophia Children's Hospital Rotterdam The Netherlands; ^6^ Department of Immunology Erasmus University MC Rotterdam The Netherlands

## Abstract

**Background:**

Mastocytosis is characterized by the accumulation of mast cells (MCs) in the skin or other organs, and can manifest at any age. A significant number of paediatric mastocytosis cases persist after puberty. In particular, monomorphic maculopapular cutaneous mastocytosis (mMPCM) is often persistent and associated with systemic mastocytosis. However, clinical differentiation of MPCM from polymorphic (p)MPCM can be difficult.

**Aim:**

To identify histopathological features that can help to distinguish mMPCM from other subtypes of paediatric mastocytosis.

**Methods:**

This was a retrospective study using skin biopsies from patients with any subtype of mastocytosis. The localization and density of the MC infiltrate, MC morphology and expression of aberrant markers were evaluated and correlated with clinical characteristics.

**Results:**

In total, 33 biopsies were available for evaluation from 26 children [(10 with mMPCM, 5 with mastocytoma, 3 with diffuse cutaneous mastocytosis (DCM), 8 with pMPCM)] and 7 adults with MPCM. The MC number was increased in all patients, but was higher in children than adults (*P* < 0.01). The presence of mMPCM was associated with sparing of the papillary dermis from MC infiltration, whereas MC density in the papillary dermis was highest in pMPCM and DCM (*P* < 0.01). The positive predictive value of the presence of a reticular MC infiltrate for mMPCM was 72.7% (95% CI 51.4–87.0), and the negative predictive value was 83.3% (95% CI 42.2–97.2). There were no relevant differences in the expression of CD2, CD25 or CD30 between the different subtypes.

**Conclusion:**

Skin histopathology might enhance the phenotypical differentiation of mMPCM from other subtypes in children, thereby increasing the accuracy of one's prognosis.

## Graphical abstract

In this study, we found that paediatric monomorphic maculopapular cutaneous mastocytosis (mMPCM) was associated with a specific histopathological pattern compared with other paediatric subtypes of mastocytosis such as polymorphic (p)MPCM. Paediatric mMPCM was characterized by a predominantly reticular situated MC infiltrate with sparing of the papillary dermis. This was similar to the pattern seen in adult‐onset MPCM; however, total MC counts in lesional skin were significantly higher in children compared with adults. Histopathology might be a useful addition to the clinical phenotype and laboratory parameters to distinguish mMPCM from pMPCM in children.

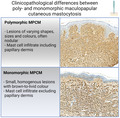

## Introduction

Mastocytosis is characterized by the accumulation of neoplastic mast cells (MCs) in tissues, most often due to an acquired activating mutation in the KIT protein, which leads to increased proliferation and survival of MCs.^1^ Mastocytosis can affect people of any age, but the clinical phenotype of childhood‐onset mastocytosis is different from that of adult‐onset mastocytosis; in children, mastocytosis is mostly restricted to the skin (cutaneous mastocytosis; CM) whereas the large majority of adult‐onset cases have systemic mastocytosis (SM).^2^ SM is diagnosed by histopathological demonstration of increased numbers of MCs in at least one extracutaneous organ, and bone marrow examination (BME) through biopsy is virtually always necessary to officially diagnose SM.^3^ The majority of adults with SM also have skin involvement.^4^

Whereas adult‐onset mastocytosis is virtually always chronic, the skin lesions in paediatric mastocytosis tend to spontaneously decrease over time before puberty, although only 6%–30% of patients reach complete resolution, according to the recent literature.^5^, ^6^, ^7^ Currently, reliable factors to predict the prognosis of childhood‐onset mastocytosis are lacking. In 2016, Hartmann *et al*.^8^ proposed a consensus classification of CM. For adults, skin involvement usually presents as a typical form of maculopapular CM (MPCM), known as monomorphic (m)MPCM, whereas several subtypes are recognized in children, ranging from single to multiple mastocytomas, diffuse CM (DCM), polymorphic MPCM (pMPCM) and mMPCM.^8^ This classification constituted a major improvement with regard to phenotyping of skin lesions, allowing for stratification of paediatric mastocytosis into different categories based on complications and prognosis. The presence of mMPCM appears to be associated with the development of chronic mastocytosis and SM in children.^9^, ^10^ However, a recent study showed a high interobserver variability for clinical classification of CM, and even experts in mastocytosis only reached an interobserver kappa rate of 0.39.^11^ In particular, the distinction of mMPCM from pMPCM can be challenging, which is a problem because of the potential prognostic implications. Histopathological characterization would thus be valuable to establish the correct diagnosis and thereby provide more accurate prognostic information.

There is a paucity of data on the histopathological features of the different subtypes of CMs. Previous studies showed a wide range of MC density in lesional skin. However, an MC count of > 75–100/mm^2^ is suggested to be abnormal, dependent on the location of the biopsy.^12^, ^13^, ^14^ Recently, an MC percentage of > 40% was also demonstrated to be specific for CM, next in specificity to the distribution of MC in skin.^15^ Expression of aberrant MC markers might also be related to certain subtypes. Whereas the expression of CD25 and/or CD30 on skin MC has been shown to correlate with the presence of SM in adults,^16^, ^17^, ^18^ expression of CD2, CD25 and CD30 could not clearly be correlated with a certain subtype of paediatric mastocytosis or with its prognosis.^19^

In this study, we aimed to identify specific histopathological features of the different subtypes of paediatric mastocytosis through a retrospective study using skin biopsies of 26 children with all subtypes of CM and 7 adults with MPCM, focusing on the distinction between mMPCM and pMPCM.

## Methods

### Patients

Patients were retrospectively included from the mastocytosis centre of a tertiary medical centre and its affiliated children's hospital. In this (inter)national referral centre for mastocytosis for all ages, all patients undergo standardized assessment. Patients for whom there was a clinical diagnosis of CM and at least one stored skin biopsy sample were eligible for inclusion. The clinical characteristics of the patients were retrospectively collected from the electronic patient records. The classification of mastocytosis in the skin as defined by Hartmann *et al*. was used to describe the type of skin lesions.^8^ The WHO definitions of CM and SM were used.^3^

### Disease classification

The cases were classified based on photographs of the skin and the description in the electronic patient records. At least two authors (SP, NA and/or MH) evaluated the subtype of CM for all patients based on photographs and description of the skin lesions in the patient files. In this classification, mMPCM was defined as small, homogeneously shaped, brown–livid, round macules, while pMPCM was defined as multiple macules, plaques or nodules of varying shape, size and/or colour. DCM was defined as widespread nodules or plaques with varying border and colours, and often with blistering, while mastocytoma was defined as one or a few, well‐demarcated, brown–livid nodular lesions with a positive Darier sign. In cases of discrepancy, the case was discussed among the authors until consensus was reached.

### Histopathological and immunochemical evaluation

See the online supplementary material for a detailed description on the histopathological evaluation (Supplementary Data S1) and antibodies used for immunohistochemistry (Supplementary Table S1).

### Statistical analysis

Statistical analysis was performed using SPSS software for Windows (V 25.0; IBM SPSS, Armonk, NY, USA) and two‐sided *P* < 0.05 was considered statistically significant. Categorical values were summarized as *n* (%), while continuous values were summarized as median with interquartile range (IQR) unless specifically stated otherwise. To compare categorical data, Fisher exact test was used, while the Mann–Whitney *U*‐test and Kruskal–Wallis test were used to compare continuous variables. Spearman rho was calculated to determine correlation between two continuous variables. For the diagnostic value of the histopathological architecture of MC infiltrate, a 2 × 2 table was used to calculate sensitivity, specificity, and positive predictive value (PPV) and negative (N)PV.

## Results

### Patient characteristics

In total, 33 patients were enrolled in the study (26 children, 7 adults). Skin biopsies were obtained between 1989 and 2020, at an age range of 0–69 years old (Table 1). Most patients (*n* = 26) had childhood onset of mastocytosis. Of the 26 children, 10 had mMPCM, 8 had pMPCM, 5 had mastocytoma and 3 had DCM. Eight children, all of whom had mMPCM, had undergone BME, which showed five to have SM according to the WHO criteria. All seven adult patients had adult‐onset mMPCM and all had undergone BME; five had indolent SM and two had isolated CM.

**Table 1:** Clinical characteristics.

	DCM (*n* = 3)	Mastocytoma (*n* = 5)	mMPCM (*n* = 10)	pMPCM (*n* = 8)	Adult‐onset (*n* = 7)	*P* ^a^
Female sex, *n* (%)	2 (66.7)	3 (60)	5 (50)	4 (50)	5 (71.4)	< 0.001
Age of onset, years; median (IQR)	0 (0–1)	0 (0–4)	0 (0–4)	0 (0–0)	30 (22–49)	N/A
Age at skin biopsy, years; median (IQR)	0 (0–1)	6 (1–7)	13 (7–31)	1 (0–3)	35 (34–51)	< 0.001
Follow‐up time since start, years; median (IQR)	3 (2–24)	4 (4–6)	25 (21–30)	8 (6–12)	7 (4–24)	0.02
Darier sign positive, *n* (%)	3 (100)	5 (100)	8 (80)	5/7 (71.4)	3/5 (60)	0.16
Anaphylaxis, *n* (%)	0	0	2 (20)	1 (12.5)	2 (28.6)	0.80
Familial mastocytosis, *n* (%)	0	0	2 (20)	0	0	0.48
Tryptase at time of skin biopsy, μg/L; median (IQR)	68.4 (17.8–119)	3.7 (3.5–3.9)	18.8 (12.7–63)	7.8 (6.1–16.4)	14.2 (5.1–35.7)	0.02
KIT *D816V* mutation detectable, *n* (%)^b^	0/2	0/1	2/6 (33.3)	1/4 (25)	5 (71.4)	0.33
Reticular localization of MC infiltrate, *n* (%)	0	4 (80)	8/9 (88.9)	3 (37.5)	7 (100)	< 0.01
Basal pigmentation, *n* (%)	0	1 (20)	5 (50)	2 (25)	6 (85.7)	0.04
Vascular ectasia, *n* (%)	0	2 (40)	6 (60)	2 (25)	6 (85.7)	< 0.05
Eosinophilia, *n* (%)	2 (66.7)	3 (60)	6 (60)	5 (62.5)	6 (87.5)	0.63
Spindle‐shaped MC in skin, *n* (%)	2 (66.7)	0	5 (50)	2 (25)	7 (100)	< 0.01
CD2 positive/negative, *n* (%)	2 (66.7)	2 (40)	7/9 (77.8)^c^	6 (76)	1 (14.3)	0.07
CD25 positive/negative, *n* (%)	0	5 (100)	6/9 (66.7)	3 (37.5)	0	0.001
CD30, *n* (%) positive/negative	2 (66.7)	5 (100)	2/9 (22.2)	3 (37.5)	2 (28.6)	0.04

DCM, diffuse cutaneous mastocytosis; IQR, interquartile range; MC, mast cell; MPCM, maculopapular cutaneous mastocytosis; mMPCM, monomorphic maculopapular cutaneous mastocytosis; pMPCM, polymorphic maculopapular cutaneous mastocytosis.

^a^ Significant *P* values are shown in bold.

^b^ D816V RQ‐PCR was performed on bone marrow in eight children and peripheral blood in four children in total, while another KIT mutation in exon 9 was found in one child with DCM.

^c^ There was too little material for immunohistochemical staining of one patient with mMPCM.

### Monomorphic maculopapular cutaneous mastocytosis is characterized by a specific dermal mast cell distribution

#### Mast cell density

**Figure 1:**
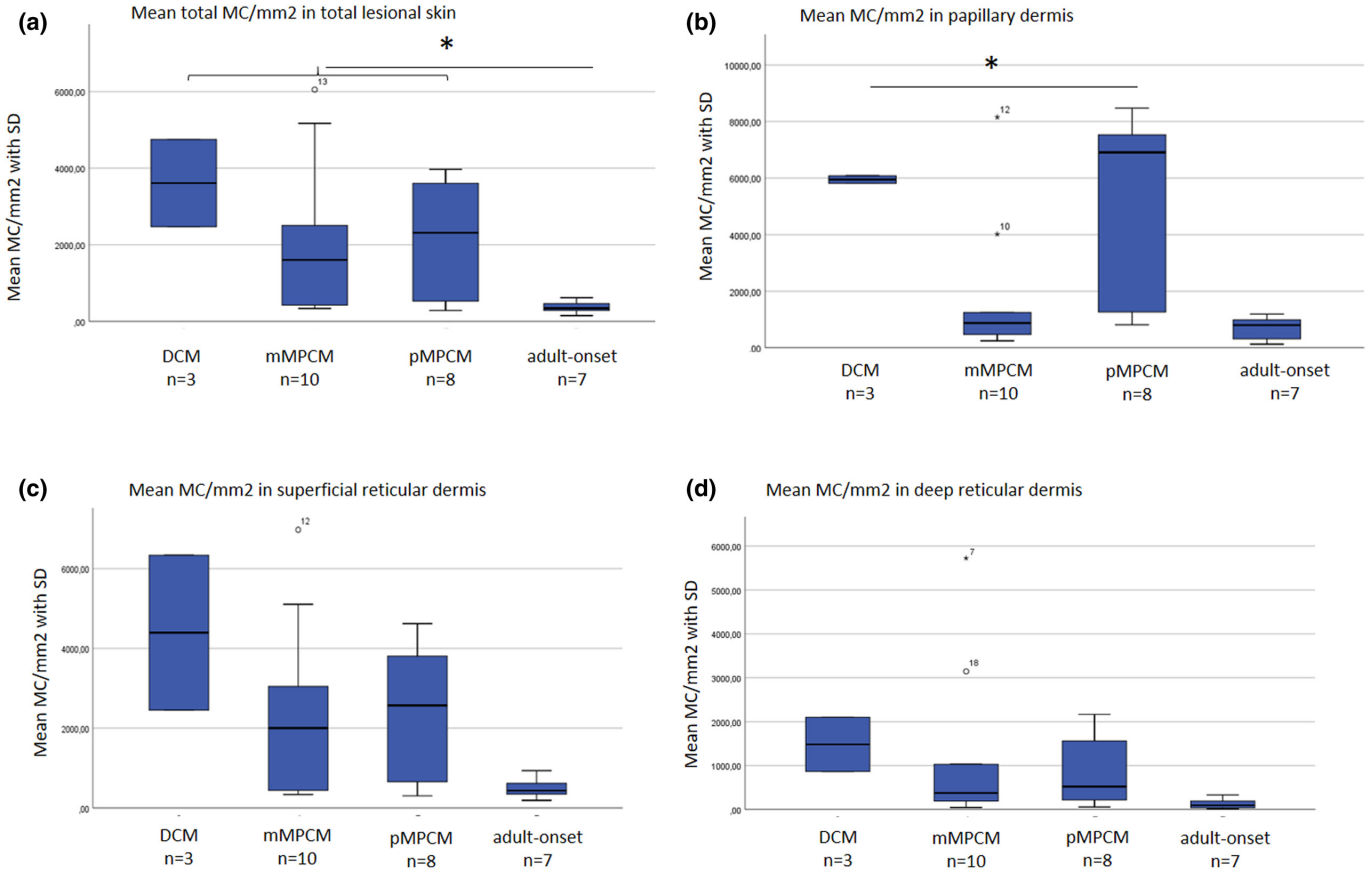
(a–d) Mean mast cell (MC) count in total skin and different skin layers for all subgroups the bars depict standard deviation. (a) There was no difference between mean total MC/mm^2^ between monomorphic maculopapular cutaneous mastocytosis (mMPCM) and polymorphic MPCM (pMPCM) in children, although the mean total MC was higher in children than adults (*P* < 0.01 for bivariate analysis). (b) The MC count was significantly lower in papillary dermis of mMPCM in children compared with other paediatric subtypes (*P* = 0.02). (c) There were no statistically significant differences in superficial reticular dermis between subtypes (*P* = 0.06). (d) The MC count was lowest in the deep reticular dermis across all subtypes, but lowest in adult patients (*P* < 0.03). DCM, diffuse cutaneous mastocytosis. [Colour figure can be viewed at wileyonlinelibrary.com]

We assessed mean total MC density and found that the total MC number was the highest in DCM, while it was comparable for both mMPCM and pMPCM (2084 MC/mm^2^ vs. 2145, respectively) (Fig. 1, Supplementary Table S2). The mean total MC count in adult skin was 374 MC/mm^2^ (*P* = 0.004 for adults vs. all children). There was a negative correlation between the age at which the skin biopsy was obtained and the MC count in skin (Spearman ρ = −0.67, *P* < 0.001). No correlation was found between the MC abundance in skin and either a history of anaphylaxis or serum tryptase levels. However, serum tryptase levels were higher in mMPCM compared with pMPCM, without reaching statistical significance (*P* = 0.20) (Supplementary Fig. S1).

#### Mast cell distribution

**Figure 2:**
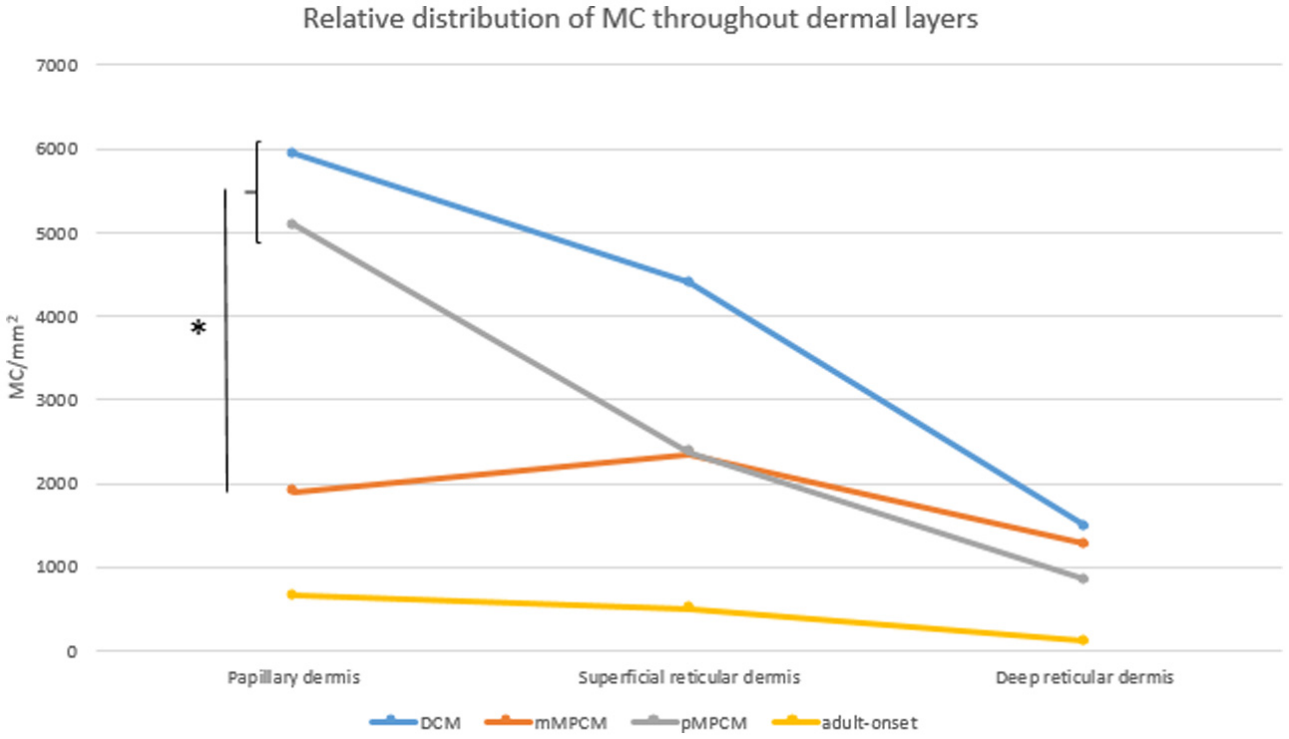
Relative distribution of mean mast cell (MC) count throughout the dermal layers. For standard deviations, see Fig. 1. *The mean MC number was significantly higher in the papillary dermis for diffuse cutaneous mastocytosis (DCM) and polymorphic maculopapular cutaneous mastocytosis (pMPCM) compared with monomorphic maculopapular cutaneous mastocytosis (mMPCM) (*P* < 0.01). [Colour figure can be viewed at wileyonlinelibrary.com]

**Figure 3:**
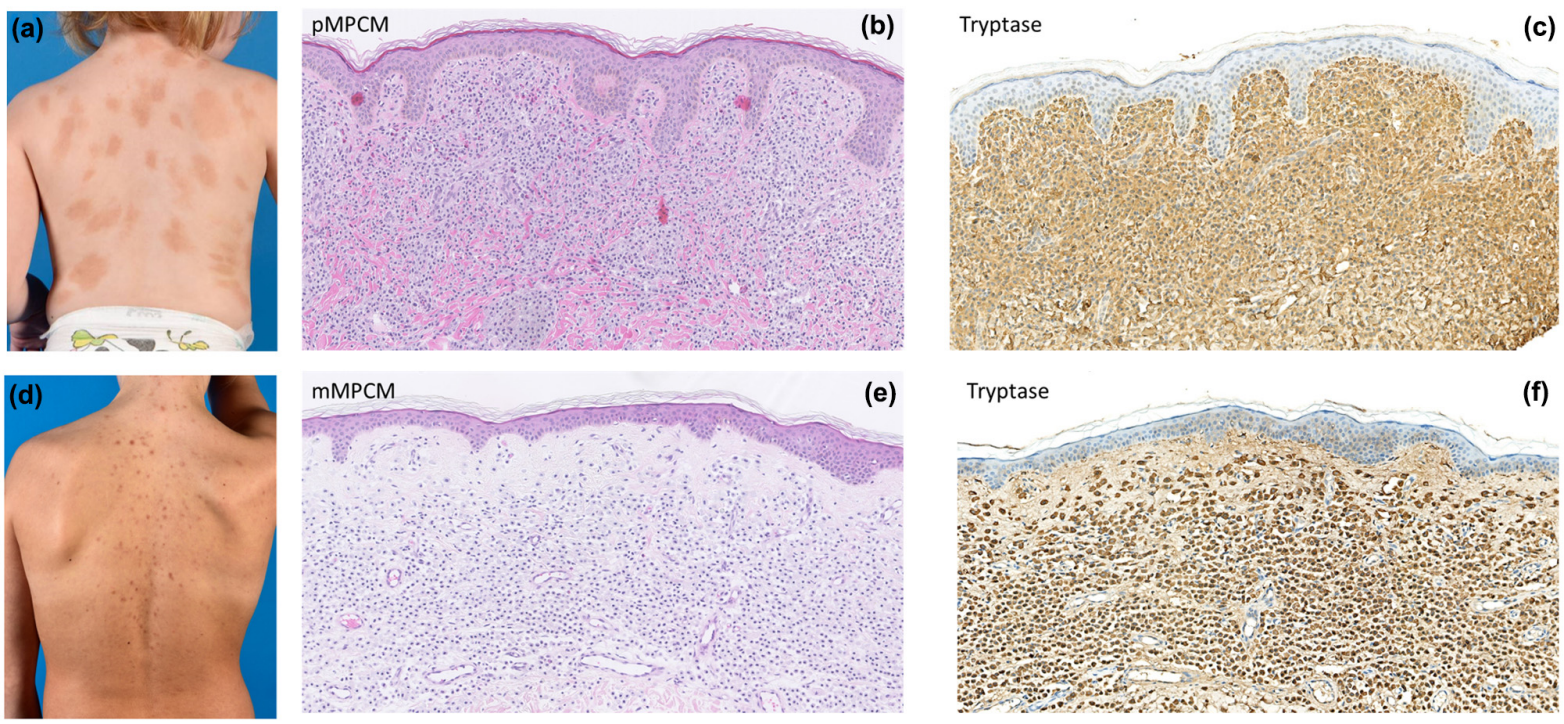
(a–f) Representative examples of the clinical phenotype and matching histopathology for monomorphic maculopapular cutaneous mastocytosis (mMPCM) and polymorphic MPCM (pMPCM). (a) A 2‐year‐old child with pMPCM, with (b) corresponding skin biopsy in showing a dense infiltrate throughout both the papillary and reticular dermis (haematoxylin and eosin stain, original magnification × 25); (c) the infiltrate was confirmed to consist of mast cells (tryptase, original magnification × 25). (d) A 10‐year‐old child with mMPCM, with (e) corresponding skin biopsy showing a dense infiltrate in the reticular dermis with papillary sparing (haematoxylin and eosin stain, original magnification × 25); (f) the infiltrate was confirmed to consist of mast cells (tryptase, original magnification × 25). [Colour figure can be viewed at wileyonlinelibrary.com]

Interestingly, clear differences were observed in the dermal distribution of the MC infiltrate between subgroups of paediatric mastocytosis (Table 2). In particular, mMPCM displayed a distinct pattern in which the papillary dermis was relatively spared from MC infiltration, whereas high MC numbers were found in the papillary dermis in pMPCM and DCM (*P* < 0.01 for comparing papillary MC count for mMPCM with the sum of DCM + pMPCM) (Fig. 2). The MC numbers in the reticular dermis were comparable for mMPCM and pMPCM (Figs 1 and 2). Figure 3 depicts two representative examples of children with mMPCM and pMPCM. It thus appears that sparing of the papillary dermis positively correlates with the presence of mMPCM compared with pMPCM or DCM.

**Table 2:** Correlation of type of skin lesions with localization of mast cell infiltrate in paediatric mastocytosis (*P* < 0.04).

Appearance^a^	mMPCM	pMPCM
Superficial reticular infiltrate with papillary sparing	8	3
Papillary‐dominant infiltrate	1	5
Total	9^b^	8

mMPCM, monomorphic maculopapular cutaneous mastocytosis; pMPCM, polymorphic maculopapular cutaneous mastocytosis.

^a^ The architecture of the mast cell infiltrate was determined by overall assessment of the skin biopsy on a low magnification.

^b^ The localization could not be determined in 1 patient with mMPCM.

### Mast cell architecture

In addition to determining mean absolute MC counts per dermal layer, the architecture of the MC infiltrate was also determined by overall assessment of the skin biopsy on a low magnification, distinguishing papillary sparing vs. papillary involvement of the infiltrate. The sensitivity of papillary sparing by infiltration for detecting mMPCM was 88.9% (95% CI 51.8–99.7) with a specificity of 62.5% (95% CI 24.5–91.5). The PPV for mMPCM was 72.7% (95% CI 51.4–87.0) and the NPV was 83.3% (95% CI 42.2–97.2). Thus, the presence of clear MC infiltration of the papillary dermis could be used to exclude mMPCM when the clinical phenotype is not entirely clear.

#### Correlation between mast cells and other characteristics

There was no correlation between the localization of the MC infiltrate and positive Darier sign, KIT *D816V* mutational status or anaphylaxis, across all subgroups. Of note, papillary sparing was also noted in mastocytoma (Supplementary Table S3); however, this represents such a distinct clinical phenotype that histopathology will usually not be necessary to aid in the exact classification.

### Similarities between paediatric monomorphic maculopapular cutaneous mastocytosis and adult‐onset maculopapular cutaneous mastocytosis

As mMPCM in children is phenotypically most similar to adult‐onset CM, we compared the histopathological features of these two groups. Although the mean MC count in the total skin and for each layer were significantly higher in paediatric mMPCM than in adult mMPCM (Figs 1 and 2), sparing of the papillary dermis was observed in most adults (5 of 7), which is similar to paediatric mMPCM (Supplementary Table S3). Furthermore, basal pigmentation and vascular ectasia were more frequent in adult‐onset MPCM and in paediatric mMPCM compared with the other paediatric subtypes (Table 1). Spindle‐shaped MCs were observed in all adults, compared with only 50% (5 of 10) of the children with mMPCM but were also seen in 25% (2 of 8) of the children with pMPCM, rendering this a less reliable variable to distinguish these subtypes based on histopathological features.

### Expression of aberrant markers by skin mast cells

**Figure 4:**
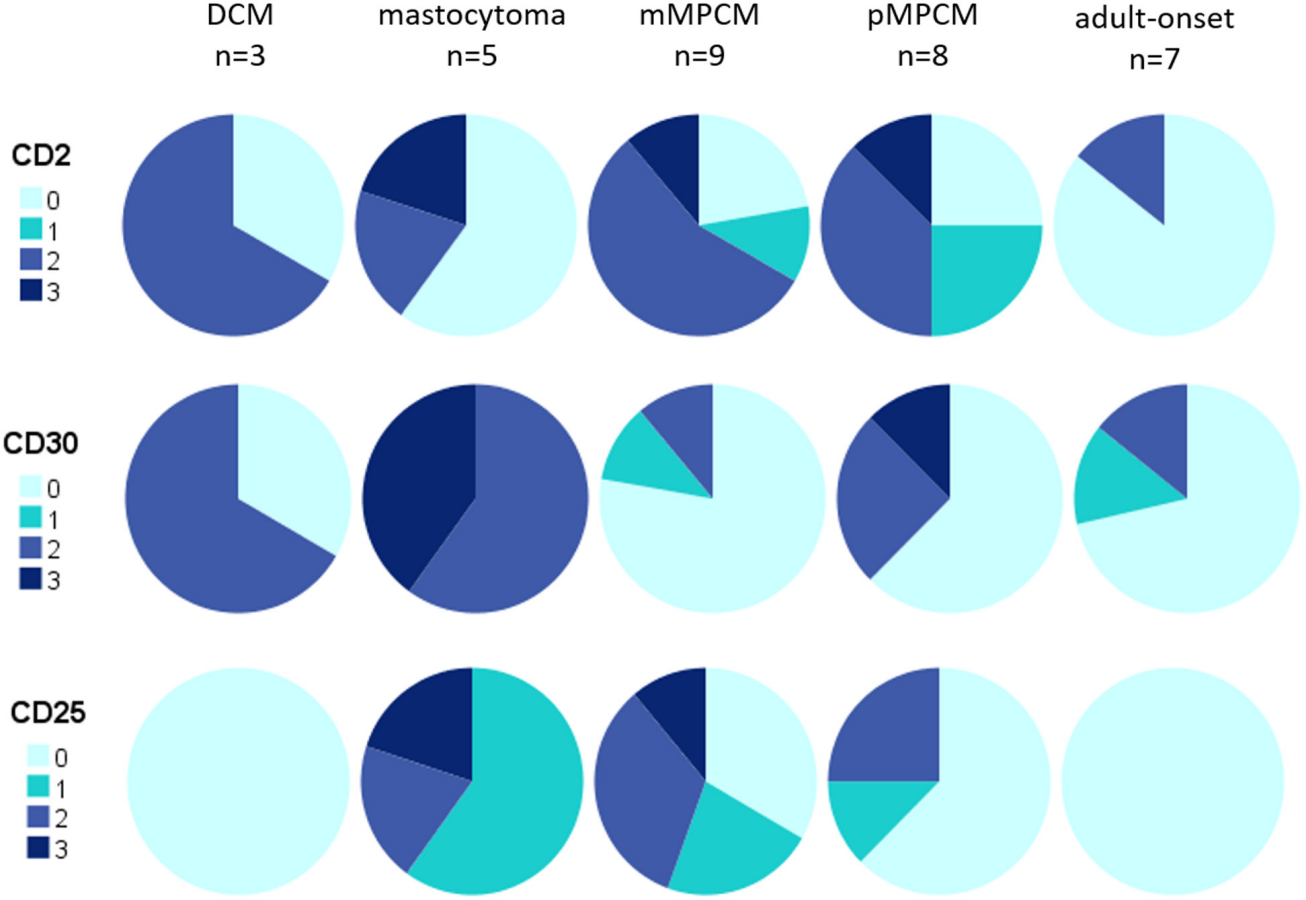
Aberrant marker expression across all subgroups. Graded expression of markers of aberrancy on skin mast cells (0 = no expression; 1 = mild; 2 = moderate; 3 = profound). DCM, diffuse cutaneous mastocytosis; mMPCM, monomorphic maculopapular cutaneous mastocytosis; pMPCM, polymorphic maculopapular cutaneous mastocytosis. No statistical significance was reached when comparing each marker expression across all five subgroups (Fisher exact test). [Colour figure can be viewed at wileyonlinelibrary.com]

The MC expression of CD117, tryptase and FcεRI was high in all 33 cases. No clear differences could be observed in the expression of CD2, CD25 or CD30 across all paediatric subgroups (Fig. 4). In children, there was also no correlation between the localization of the MC infiltrate and expression of CD2, CD25 or CD30. In the adult patients, little to no expression of any aberrant marker was detected (Fig. 4). When comparing the marker expression graded from 0 to 3 for intensity across all subgroups, no statistical significance was found (Fisher exact test), possibly due to the small subgroup sizes. Although the distribution of the MC infiltrate throughout the dermis was similar in paediatric mMPCM compared with adult‐onset MPCM as described above, the marker expression was different between these two groups (Fig. 4), but the subgroups were considered too small for statistical analysis. Of note, a granular pattern of expression in skin, rather than a membrane‐bound pattern, was seen for 100% of patients with CD2‐positive MCs, 46% of those with CD25‐positive MCs and 28% of those with CD30‐positive MCs.

## Discussion

In this study, we found that paediatric mMPCM was associated with a specific histopathological pattern in which the papillary dermal layer was relatively spared from MC infiltration, in contrast to a predominantly papillary infiltrate in pMPCM and DCM. Additionally, we found that the papillary dermis was also often spared in adult‐onset MPCM, corroborating the notion that mMPCM in children is phenotypically similar to adult‐onset MPCM, although the small subgroup numbers prevented definitive conclusions on these similarities. Because not all children in our cohort underwent BME, conclusions on the association between histopathological features and the presence of SM also pose a risk of bias and should be investigated in a prospective study. Three of the eight children with typical pMPCM also displayed a pattern of relative papillary sparing from MC infiltration. One of these children has now reached adulthood and has persisting cutaneous disease, with detectable *D816V* mutation in the peripheral blood, but she declined biopsy for BME. Although this is anecdotal evidence, it might underline the association between a reticular MC infiltrate and persistent disease, in addition to the clinical phenotype. A longer follow‐up time, allowing for the rest of the cohort to reach adulthood, is necessary to confirm a potential association between histopathology and prognosis.

Since the establishment of the classification of CM by Hartmann *et al*.^8^ in 2016, it has become clear that the type of skin lesions correlate with prognosis in paediatric mastocytosis.^20^, ^21^ Therefore, this new classification is an important tool to phenotype children with mastocytosis. However, distinguishing mMPCM from pMPCM can be difficult in clinical practice, as was demonstrated by a recent paper that found an interobserver kappa rate of 0.17 between general dermatologists and 0.39 among 10 experts.^22^ A combination of clinical phenotype, laboratory parameters and MC distribution in skin could thus potentially identify those children at risk for SM, thereby supporting decisions on additional diagnostic examinations and follow‐up. KIT mutation status can also be taken into account, as presence of the *D816V* mutation is linked to SM in children.^23^, ^24^ However, none of the previous studies on this topic made a distinction between mMPCM and pMPCM. Owing to the retrospective nature of our study, the KIT *D816V* mutation status was not known for all patients, preventing conclusions on its association with the subtype of paediatric mastocytosis.

When we assessed previously described aberrant lymphoid markers on skin MC, the expression patterns did not correlate with certain paediatric subtypes in our cohort. Previous studies on this topic also found no correlation between expression of CD2 and CD25 and the clinical course of paediatric mastocytosis, although no publications differentiated mMPCM from pMPCM.^18^, ^19^ We found low expression of CD2, CD25 and CD30 on skin MC in adults, which was unexpected and is in contrast to previous studies showing CD30 positivity up to 92.8% of patients with SM^17^ and CD25 expression in 100%.^16^ Because only seven adults were included in our study, detection bias might play a role, and no definitive conclusions on the value of these markers can be drawn here.

This study has some limitations. Firstly, the subgroups were too small to reliably correlate the clinical subtype of skin lesions to clinical characteristics or the presence of SM. Furthermore, skin biopsies were obtained at varying ages due to the retrospective nature of the study, which might theoretically affect the mean total MC count and distribution. However, our results proved to be fairly uniform within subgroups, suggesting that the histopathological features are stable throughout a patient's life. Lastly, conclusions with regard to the prognosis could not be made because not all patients have yet reached adulthood, and BME was not routinely performed.

## Conclusion

Paediatric mMPCM was associated with a specific histopathological pattern compared with other paediatric subtypes of mastocytosis, characterized by relative sparing of the papillary dermis from MC infiltration. However, children had significantly higher total MC counts in lesional skin compared with adults. No relevant differences were observed regarding the expression of CD2, CD25 or CD30 between paediatric subtypes. Histopathology could be a useful addition to the clinical phenotype and laboratory parameters to distinguish mMPCM from pMPCM in children. A prospective study in a larger cohort is necessary to answer the questions emerging from the current data.

**What's already known about this topic?**

- Paediatric mastocytosis is always associated with cutaneous involvement.
- Various subtypes are recognized, each with their own clinical characteristics.
- The presence of mMPCM in children appears to be associated with persistent disease and development of SM.
- However, distinguishing mMPCM from pMPCM can be difficult in clinical practice.

**What does this study add?**

- We found that mMPCM displays a distinct histopathological pattern, in which the MC infiltrate is mainly situated in the reticular dermis with sparing of the papillary dermis.
- This is in contrast to pMPCM and DCM, in which the MC number was found to be highest in the papillary dermis.
- Histopathological evaluation of lesional skin can thus be a feasible instrument to distinguish mMPCM from other subtypes of paediatric mastocytosis.

## Conflict of interest

The authors declare that they have no conflict of interest.

## Funding

None.

## Ethics statement

This study was conducted according to the latest Declaration of Helsinki guidelines. Approval from a medical ethics committee and informed consent of participants was waived because all data were gathered retrospectively and anonymously. Written informed consent was obtained from the parents of the two children whose images were used.

## Data availability

Data are available on request from the corresponding author.

## Supporting information


**Data S1.** Methods.
**Table S1.** Description of antibodies used for immunohistochemistry.
**Table S2.** Mean total mast cell (MC) per mm^2^ in different skin layers for paediatric vs. adult‐onset mastocytosis.
**Table S3.** Pattern of mast cell (MC) infiltrate: presence or absence of papillary sparing as determined on low magnification view of skin biopsies, per mastocytosis subtype. Absolute numbers are given.

**Figure S1** Mean serum tryptase levels at the time of skin biopsy correlated to subgroup. *The mean serum tryptase levels at the time of skin biopsy were higher in children with diffuse cutaneous mastocytosis (DCM) than other subtypes (*P* < 0.02). There was a trend towards higher tryptase levels in monomorphic maculopapular cutaneous mastocytosis (mMPCM) compared with polymorphic maculopapular cutaneous mastocytosis (pMPCM), but this did not reach statistical significance (*P* = 0.20).
