# Supplementary material for: Histopathological characteristics are instrumental to distinguish monomorphic from polymorphic maculopapular cutaneous mastocytosis in children
Source: Clin Exp Dermatol. 2022 Jul 11;47(9):1694–702. doi: 10.1111/ced.15262 (PMC9544455; doi:10.1111/ced.15262)
Supplement: Supplementary file 1 — Data S1. Methods. Table S1. Description of antibodies used for immunohistochemistry. Table S2. Mean total mast cell (MC) per mm2 in different skin layers for paediatric vs. adult‐onset mastocytosis. Table S3. Pattern of mast cell (MC) infiltrate: presence or absence of papillary sparing as determined on low magnification view of skin biopsies, per mastocytosis subtype. Absolute numbers are given. [file CED-47-1694-s001.docx]

Supporting material belonging to

**Histopathological characteristics are instrumental to distinguish monomorphic from polymorphic maculopapulous cutaneous mastocytosis in children.**

**Methods**

*Histopathological evaluation*

Formalin-fixed paraffin embedded (FFPE) tissue sections were stained with hematoxylin and eosin for light microscopic evaluation. Histopathological features were reviewed by two independent dermatopathologists (J.D. and A.M.) by routine light microscopy (Table 2) for the following parameters:

1. Dominant inflammatory architectural pattern on low magnification:
   1. MC infiltrate predominantly situated in the papillary dermis
   2. MC infiltrate predominantly situated in the reticular dermis with sparing of the papillary dermis
2. detailed inflammatory architectural pattern on high magnification
   1. perivascular and/or interstitial,
   2. papillary dermis and/or superficial/mid/deep reticular dermis)
3. perivascular and/or interstitial inflammatory cell type (mast cells, lymphocytes, histiocytes, plasma cells, eosinophils, neutrophils)
4. mast cell shape (oval shape, spindle shaped, or pleomorphic or bilobated nuclei)
5. basal pigmentation and vascular ectasia.

The inflammatory infiltrate was scored on a nominal scale of 0-3: none (0), mild (1), moderate (2), profound (3). All other histopathological parameters were scored dichotomously (present/absent). Abnormal MC shape was found to be present when >5% of cells were aberrantly shaped.

*Immunohistochemistry and mast cell count*

All skin biopsies were stained for tryptase, CD117, CD2, CD30, CD25 and FCεR1G performed by an automated staining method using the Ventana Benchmark Discovery (Ventana Medical Systems Inc.) (Table S1). In brief, wet slides were loaded and incubated with primary antibody of interest for 32 minutes (Table 1) at 37˚C followed by detection with either omnimap-anti rabbit or mouse, labeled with HRP. Mast cell density per mm^2^ was performed on tryptase stained slides. Mean tryptase positive mast cells were calculated from two representative stained sections of good quality on the same slide. Mast cells were counted in three different layers of the dermis: papillary dermis, superficial reticular dermis (surrounding the superficial vascular plexus) and mid-deep reticular dermis. Mast cells were counted after scanning the slide using a Hamamatsu slide scanner and NDPviewi software. After digitalization, the papillary dermis was annotated and the surface calculated. Next, three contiguous horizontal areas of 0,238 mm^2^ (similar to a 400x magnification (1 HPF) on an Olympus BX40 microscope) were selected in the superficial reticular dermis (surrounding the superficial vascular plexus) and two in the mid-deep reticular dermis. Areas adjacent to the biopsy edge and around dermal nerves and adnexae were avoided during counting. Mast cells without a nucleus were only counted when 5 or more granules were counted within the cytoplasm. Mast cells were counted using a grid overlay from the grid plugin tool in IMAGEJ software. When mast cells were out of focus on the digitalized slides due to their granular appearance, exactly the same area was counted under the microscope. Mean mast cell count per mm^2^ was calculated for each dermal layer and for the whole section. The MC number was not counted in mastocytoma since those biopsies had a very high MC count throughout the dermis and this clinical entity is easily recognizable. Regarding immunohistochemistry, the intensity of CD2, CD25 and CD30 staining was graded as: negative (0), weak (1), intermediate (2) and strong (3).

| **Supplementary table 1.**  Description of antibodies used for immunohistochemistry. | | | |
| --- | --- | --- | --- |
| **Antibody** | **Host and targets species** | **Supplier** | **Concentration/dilution** |
| Tryptase | Monoclonal mouse-anti-human (clone G3) #342M-18 | Cell Marque | 0.05 µg/ml |
| CD117 | monoclonal rabbit-anti-human (clone 4R145) #117R-16 | Cell Marque | 10,9 µg/ml |
| CD2 | monoclonal mouse-anti-human (clone MRQ-11) #760-4377 | Cell Marque | 0,75 µg/ml |
| CD30 | monoclonal mouse-anti-human (clone BERH2) #790-4858 | Ventana | 123 µg/ml |
| CD25 | monoclonal mouse-anti-human (clone 4C9) #760-4439 | Ventana | 0,22 µg/ml |
| FCER1G | Polyclonal-rabbit-anti human FCER1G, HPA026872 | Sigma | 1/100 |

| **Supplementary table 2.**  Mean total MC/mm^2^ in different skin layers for pediatric versus adult-onset mastocytosis. | | | |
| --- | --- | --- | --- |
|  | Pediatric mastocytosis (n=26) | Adult-onset mastocytosis (n=7) | *p value** |
| MC/mm^2^ in total skin, mean with SEM | 2151 (398) | 374 (60) | 0.004 |
| MC/mm^2^ in papillary skin, mean with SEM | 2667 (740) | 672 (158) | 0.025 |
| MC/mm^2^ in superficial reticular skin, mean with SEM | 2576 (482) | 500 (97) | 0.15 |
| MC/mm^2^ in deep reticular skin, mean with SEM | 1116 (324) | 129 (48) | 0.004 |
| SEM= standard error of the mean ; *calculated by Mann Whitney U | | | |

| **Supplementary table 3.**  Pattern of mast cell (MC) infiltrate: presence or absence of papillary sparing as determined on low magnification view of skin biopsies, per mastocytosis subtype. Absolute numbers are given. | | | | | |
| --- | --- | --- | --- | --- | --- |
|  | DCM  n=3 | Mastocytoma  n=5 | mMPCM  n=9 | pMPCM  n=8 | Adult-onset mastocytosis  n=7 |
| Relative sparing of papillary dermis from MC infiltration | 0 | 4 | 8 | 3 | 5 |
| MC infiltration present in papillary dermis | 3 | 1 | 1 | 5 | 2 |
| DCM: diffuse cutaneous mastocytoma; mMPCM: monomorphic maculopapular cutaneous mastocytosis; pMPCM: polymorphic MPCM. The architectural pattern was graded by overall assessment on low microscopic magnification by 2 experienced dermatopathologists. | | | | | |

**Figure legends**

**Figure S1. Mean serum tryptase levels at the time of skin biopsy correlated to subgroup.**

*****The mean serum tryptase levels at the time of skin biopsy were higher in children with DCM than other subtypes (p 0.024). There was a trend towards higher tryptase levels in mMPCM compared with pMPCM without reaching statistical significance (p 0.204). DCM = diffuse cutaneous mastocytosis, mMPCM = monomorphic maculopapular cutaneous mastocytosis, pMPCM = polymorphic maculopapular cutaneous mastocytosis.
