# Supplementary figures and images for: Histopathological characteristics are instrumental to distinguish monomorphic from polymorphic maculopapular cutaneous mastocytosis in children
Source: Clin Exp Dermatol. 2022 Jul 11;47(9):1694–702. doi: 10.1111/ced.15262 (PMC9544455; doi:10.1111/ced.15262)

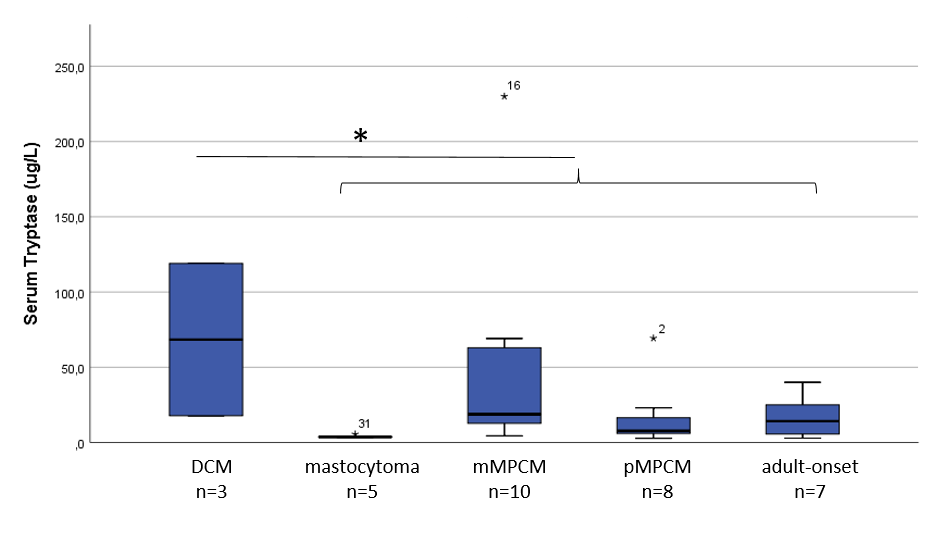

Supplement: Supplementary file 2 — Figure S1 Mean serum tryptase levels at the time of skin biopsy correlated to subgroup. *The mean serum tryptase levels at the time of skin biopsy were higher in children with diffuse cutaneous mastocytosis (DCM) than other subtypes (P < 0.02). There was a trend towards higher tryptase levels in monomorphic maculopapular cutaneous mastocytosis (mMPCM) compared with polymorphic maculopapular cutaneous mastocytosis (pMPCM), but this did not reach statistical significance (P = 0.20). [file CED-47-1694-s002.tif]
